# Transcriptomic analysis of Arabidopsis developing stems: a close-up on cell wall genes

**DOI:** 10.1186/1471-2229-9-6

**Published:** 2009-01-16

**Authors:** Zoran Minic, Elisabeth Jamet, Hélène San-Clemente, Sandra Pelletier, Jean-Pierre Renou, Christophe Rihouey, Denis PO Okinyo, Caroline Proux, Patrice Lerouge, Lise Jouanin

**Affiliations:** 1 Department of Chemistry, University of Saskatchewan, 110 Science Place, Saskatoon, SK S7N 5C9, Canada; 2 Laboratoire de Biologie Cellulaire, Institut National de la Recherche Agronomique (INRA), Route de St-Cyr, 78026 Versailles Cedex, France; 3 Surfaces Cellulaires et Signalisation chez les Végétaux, UMR 5546 CNRS-UPS, Université de Toulouse, 24 Chemin de Borde Rouge, BP42617, 31326-Castanet-Tolosan, France; 4 Unité de Recherche en Génomique Végétale, UMR INRA 1165-CNRS 8114, UEVE, 91057 Evry cedex, France; 5 Faculté des Sciences, FRE CNRS 3090, IFRMP23, Université de Rouen, F-76821 Mont Saint Aignan Cedex, France

## Abstract

**Background:**

Different strategies (genetics, biochemistry, and proteomics) can be used to study proteins involved in cell biogenesis. The availability of the complete sequences of several plant genomes allowed the development of transcriptomic studies. Although the expression patterns of some *Arabidopsis thaliana *genes involved in cell wall biogenesis were identified at different physiological stages, detailed microarray analysis of plant cell wall genes has not been performed on any plant tissues. Using transcriptomic and bioinformatic tools, we studied the regulation of cell wall genes in *Arabidopsis *stems, *i.e. *genes encoding proteins involved in cell wall biogenesis and genes encoding secreted proteins.

**Results:**

Transcriptomic analyses of stems were performed at three different developmental stages, *i.e.*, young stems, intermediate stage, and mature stems. Many genes involved in the synthesis of cell wall components such as polysaccharides and monolignols were identified. A total of 345 genes encoding predicted secreted proteins with moderate or high level of transcripts were analyzed in details. The encoded proteins were distributed into 8 classes, based on the presence of predicted functional domains. Proteins acting on carbohydrates and proteins of unknown function constituted the two most abundant classes. Other proteins were proteases, oxido-reductases, proteins with interacting domains, proteins involved in signalling, and structural proteins. Particularly high levels of expression were established for genes encoding pectin methylesterases, germin-like proteins, arabinogalactan proteins, fasciclin-like arabinogalactan proteins, and structural proteins. Finally, the results of this transcriptomic analyses were compared with those obtained through a cell wall proteomic analysis from the same material. Only a small proportion of genes identified by previous proteomic analyses were identified by transcriptomics. Conversely, only a few proteins encoded by genes having moderate or high level of transcripts were identified by proteomics.

**Conclusion:**

Analysis of the genes predicted to encode cell wall proteins revealed that about 345 genes had moderate or high levels of transcripts. Among them, we identified many new genes possibly involved in cell wall biogenesis. The discrepancies observed between results of this transcriptomic study and a previous proteomic study on the same material revealed post-transcriptional mechanisms of regulation of expression of genes encoding cell wall proteins.

## Background

Plant stems represent a major contribution to crop biomass. They contain a large proportion of cell walls [1]. Cell walls play different roles in growth, development and transportation of nutrients and water. They also provide a mechanical support for plants as well as an efficient protection against environmental stresses [2-4].

Cell walls mainly consist of large biopolymers such as cellulose, hemicelluloses, pectins and lignins. The structure and composition of these polymers vary during development not only between different tissues of the same plant, but also from one plant to another [5,6]. Although plant cell walls are mainly composed of carbohydrates and lignins, cell wall proteins (CWPs) make up approximately 10% of their mass. CWPs can become *N*- and/or *O*-glycosylated during their transit through the secretory pathway [7]. A recent proteomic analysis using ConA sepharose affinity chromatography on *Arabidopsis *stems showed that the majority of the trapped proteins were predicted to be secreted [8]. In addition, some CWPs receive a glycosylphosphatidylinositol (GPI) anchor [9].

Classical approaches for the study and identification of CWPs are based on cell wall purification, protein extraction, separation by electrophoresis and identification of proteins by mass spectrometry. Many proteomic analyses were performed on *Arabidopsis *cell suspension cultures or organs including roots, stems, leaves and etiolated hypocotyls [8,10-14]. To better understand the biological processes encompassed by the proteins identified by proteomic approaches, CWPs were classified according to their known or predicted functions [15].

However, several problems are encountered during extraction, detection and identification of CWPs by proteomic approaches [16]. It means that all available cell wall proteomes are actually sub-proteomes. On the other hand, they only provide information on extracellular proteins. All the intracellular proteins as well as many membrane proteins contributing to cell wall biogenesis are not taken into consideration.

The availability of the complete sequences of several plant genomes allowed the development of transcriptomic studies [17]. Global gene expression as well as regulation at different physiological stages or in response to changes in the environment can be analyzed. The expression patterns of *Arabidopsis *genes that are supposed to be involved in cell wall dynamics were analyzed [18]. Molecular events involved in the transition from primary to secondary growth were studied in *Arabidopsis *and hybrid aspen trees [19-23]. Genes encoding proteins involved in the synthesis of cell wall components, cell death proteins, transporters, cytoskeleton-interacting proteins, and transcription factors were identified. Finally, the expression of over 1600 genes encoding carbohydrate-active enzymes (CAZy) was analyzed in *Populus trichocarpa *[24].

This work describes the identification of *Arabidopsis *genes expressed at different developmental stages of stems. The aims were (i) to get an overview of the transcriptional activity of genes possibly involved in cell wall biogenesis and of genes encoding cell wall proteins; (ii) to compare the results from this transcriptome analysis to those of a previous proteomic analysis performed on the same material [8]. New genes that are possibly involved in cell wall biogenesis during stem elongation were identified and the importance of post-transcriptional events in regulation of genes encoding CWPs was shown.

## Results and discussion

In order to analyse the expression pattern of genes encoding CWPs, Complete *Arabidopsis *Transcriptome MicroArrays (CATMAs) were used [25,26]. CATMA contains 24,576 Gene Specific Tags (GSTs) for known or predicted genes. Three developmental stages of stems were studied: young elongating stems, intermediate stage, and mature stems as described in Methods. In stems, two biological processes are of high importance [27]. Cell elongation and synthesis of primary cell walls occur until the final size of the stems is reached. At the same time, differentiation of conducting tissues occurs. Some vessels at the bottom of young stems already have thickened cell walls and lignin deposition is starting. First signs of fiber differentiation appear at the intermediate stage while thickened secondary cell walls undergo lignification. In both cases, synthesis of cell wall components as well as their rearrangements are major events.

### 1. Sugar composition of stems at various stages of development

**Figure 1:**
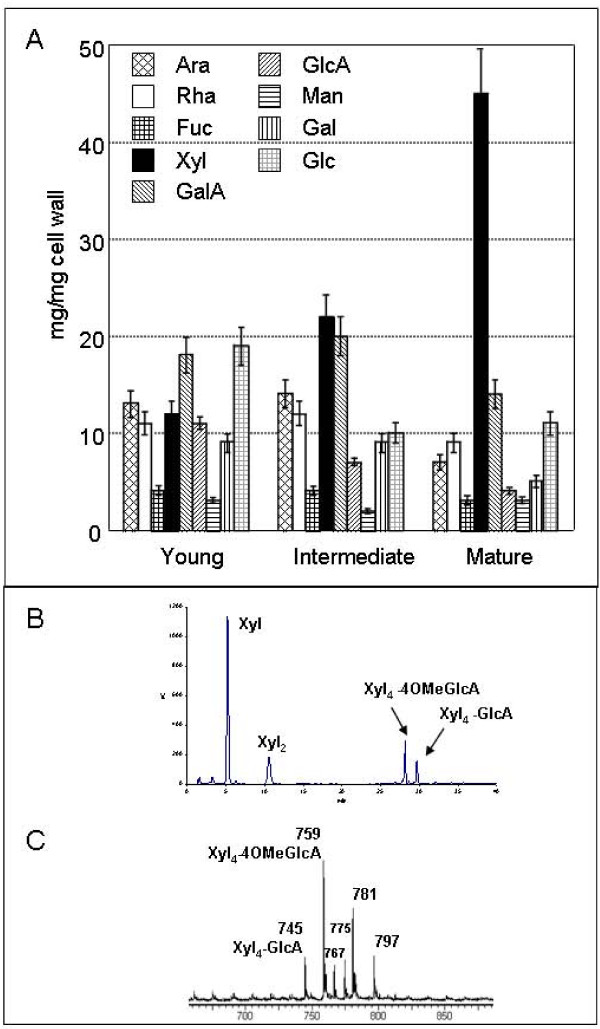
**Monosaccharide composition of cell walls from three development stages: young, intermediate and mature stems**. **A**: Monosaccharide composition was determined as described in Methods. **B**: HPAEC elution profile of the endo-xylanase-generated fragments obtained from alcali-extracted polymer of *Arabidopsis *stems. **C**: MALDI-TOF mass spectrum of acidic oligoxylan products generated by the endo-xylanase digestion of the alkali-extracted polylmer of *Arabidopsis *stems.

Cell wall polysaccharide biogenesis includes polymer synthesis, secretion, assembly, and rearrangement during development [25-28]. Consequently, the sugar composition of the cell wall polysaccharides is in relation with development stage of the cells. For this reason analysis of sugar composition of cell wall materials from stems of three developmental stages were performed (Figure 1A). This analysis was performed as previously described [29]. The results revealed a drastic increase in Xyl amount from young elongating to mature stems. Little or no significant changes in the amounts of the other sugars were observed. These results strongly suggested that xylan is mostly deposited at intermediate and mature stages of stem development.

In order to confirm the presence of xylan and to determine its structure, additional analyses were performed. The alcohol-insoluble material from *Arabidopsis *mature stems was sequentially extracted with ammonium oxalate, 1 M and 4 M KOH. The glycosidic composition of these three fractions was determined by gas chromatography. Analysis of the ammonium oxalate extract revealed the presence of GalA, Ara, Rha, and Gal suggesting that this fraction was mainly composed of pectic material. In contrast, Xyl was the main monosaccharide (> 90%) of the 1 M and 4 M KOH extracts, together with small amount of Glc and traces of other sugars including GlcA and 4-*O*-methyl glucuronic acid (4OMeGlcA), indicating that xylan was the main alkali-extracted polymer of *Arabidopsis *stems.

To investigate the structure of xylan present in the alkali extracts, the fractions were treated with an endo-xylanase and the resulting fragments were analysed by High Performance Anion Exchange Chromatography-Pulse Amperometric Detection (HPAEC-PAD) and Matrix-Assisted Laser Desorption Ionization-Time of Flight mass spectrometry (MALDI-TOF MS). Similar data were obtained for 1 M and 4 M KOH extracts. The HPAEC elution profile of the endo-xylanase-generated fragments indicated the presence of Xyl and xylobiose (Xyl_2_), as well as peaks arising from xylan-derived acidic oligosaccharides that are eluted with high concentration of sodium acetate (Figure 1B). For structural identification of the acidic fragments, the endo-xylanase-generated oligomers were analysed by MALDI-TOF MS (Figure 1C). The main ion at *m/z *759 corresponded to the [M+Na]^+ ^adduct of Xyl_4_-4OMeGlcA. The ion at *m/z *745 was assigned to the [M+Na]^+ ^adduct of Xyl_4_-GlcA. Other ions corresponded to [M+2Na-H]^+ ^and [M+Na+K-H]^+ ^adducts of the two acidic xylan fragments. These data are in agreement with recent results reported in papers describing the biosynthesis of *Arabidopsis *xylan [30,31]. Acidic fragments were isolated and were analysed by proton Nuclear Magnetic Resonance (NMR, not illustrated). By comparison with previous NMR data on glucuronoxylan fragments [30-32], the spectrum was fully in agreement with the presence of a mixture of a β(1–4)-linked xylopyranose residues regularly substituted on C-2 by α-4OMeGlcA or α-GlcA in a 2/1 ratio (data not shown). The Xyl to uronic acid ratio was estimated to be about 6–7 on the basis of the peak integration of the HPAEC profile of endo-xylanase-generated oligomers (Figure 1A). In conclusion, these data indicated that a glucuronoxylan exhibiting a 4OMeGlcA/α-GlcA in a ratio of 2/1 is deposited during *Arabidopis *stem development and along with cellulose and lignin represents one of the main polymers in the mature stem.

### 2. Transcriptome microarrays analyses

In order to analyse the expression pattern of genes encoding CWPs, Complete *Arabidopsis *Transcriptome MicroArrays (CATMAs) were used [33,34]. CATMA contains 24,576 Gene Specific Tags (GSTs) for known or predicted genes. Results are expressed as log_2 _of signal mean intensity. Expression level is considered as high when values are higher than 10, moderate for values between 9 and 10 and low for values between 9 and background level estimated at 6.42 (see Methods). In young stems, 6, 6 and 48% of the probes give signals corresponding to high, moderate and low levels of transcripts, respectively; whereas 39% of the probes give a signal lower than background. In mature stems, 5, 4 and 48% of the probes give signals corresponding to high, moderate and low levels of transcripts, respectively; whereas 42% of the probes give a signal lower than background. A modulation of level of transcripts is revealed with 2313 probes at least at one of the three stages of development. Among those, 1282 probes reveal changes during the transition from young stems to intermediate stage, and 1395 probes during the transition from intermediate stage to mature stems. This transcriptome has been analyzed in more details and is described in the following sections.

#### 2.1. Levels of transcripts of genes involved in cell wall biogenesis and of genes encoding secreted proteins during stem development

The levels of transcripts of two groups of genes involved in the synthesis of cell wall components were analyzed. These genes encode intracellular proteins or proteins located at the plasma membrane (cellulose synthases). First group comprises genes encoding glycosyl transferases (GTs) involved in the synthesis of cell wall polysaccharides (see Additional file 1). Genes from all GT families analyzed had detectable levels of transcripts in all 3 samples. The levels of transcripts of only a few genes were modulated during stem development, *i.e. *20 out of 101. Most remarkable changes were noted for genes encoding cellulose synthases related to secondary wall formation (*CesA4*, *IRX5*, *At5g44030*; *CesA7*, *IRX3*, *At5g17420*) and callose synthases (*AtGSL6*, *CALS1*, *At1g05570*; *AtGSL3*, *At2g31960*). The second group comprises genes encoding proteins putatively involved in the biosynthesis of monolignols that are lignin precursors (see Additional file 2). A greater proportion of genes had modulated levels of transcripts: either a decrease during the transition from young elongating stems to intermediate stage (*PAL4*, *4CL-like1*, *CCR1*, *CAD1*, *CAD6*, *CAD7*), or an increase during transitions from young elongating stems to intermediate stage (*4CL2*, *CCoAOMT7*), or intermediate stage to mature stems (*PAL1*, *PAL3*, *4CH*, *4CL2*, *4CL-like1*, *CCoAOMT3*, *CCoAOMT7*). This analysis revealed that synthesis of the cell wall components required for biogenesis of primary and secondary walls is an active process throughout stem development.

Genes encoding secreted proteins were sorted using bioinformatics softwares as described in Methods. Genes expressed at moderate or high level have been further annotated for experimentally proven or predicted biological functions. A total of 345 genes were classified into 8 functional categories according to Jamet et al. [15] (Table 1, see Additional file 3). A large proportion of the genes encode proteins with unknown functions (34.5%, 119 genes), miscellaneous functions (17.1%, 59 genes) and proteins acting on carbohydrates (14.8%, 51 genes). Other genes encode proteins predicted to be involved in signalling (11.6%, 40 genes), proteases (7%, 24 genes), proteins with interaction domains (6.7%, 23 genes), oxido-reductases (4.6%, 16 genes), and structural proteins (3.8%, 13 genes).

**Table 1:** Repartition of genes encoding secreted proteins and having high or moderate levels of transcripts during stem development in functional classes.

**Functional classes**	**Percentage of genes**
Proteins acting on carbohydrates	14.6
Oxido-reductases	4.6
Proteins with interaction domains	6.7
Proteins involved in signaling	11.6
Proteases	7.0
Structural proteins	3.8
Miscellaneous proteins	17.1
Proteins of unknown function	34.5

Bioinformatic softwares were used to search for functional domains in the encoded proteins. The proteins were distributed in eight functional classes. Percentages of genes in each class are indicated

**Figure 2:**
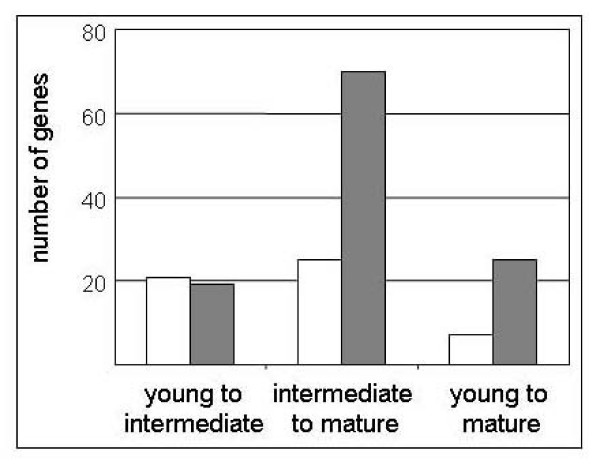
**Genes encoding secreted proteins having modulated level of expression during stem development**. Three transitions were analyzed: from young to intermediate stage of development, from intermediate to mature, and from young to mature. Numbers of genes that are up-regulated and down-regulated during these transitions are represented by white and grey bars respectively. Data are shown in Additional file 3.

Overall and as shown in Figure 2, this transcriptome analysis revealed that 21 genes were up-regulated during the transition from young stems to intermediate stage whereas 25 genes were up-regulated during the transition from intermediate stage to mature stems. On the contrary, 19 genes were down-regulated during the transition from young stems to intermediate stage, whereas 70 genes were down-regulated during the transition from intermediate stage to mature stems. Finally, 7 genes were up-regulated throughout the development of stems whereas 25 genes were down-regulated. All these changes probably correspond to major changes in cell walls during stem development either through rearrangements of polysaccharide networks or other biological processes not yet described.

#### 2.2. Genes involved in cell wall biogenesis

Genes involved in cell wall biogenesis *per se *encode proteins that can be intracellular, extracellular, or located at the plasma membrane. Intracellular proteins are those performing reactions necessary for the synthesis of all cell wall polysaccharides except cellulose and monolignols. Cellulose and callose synthases are at the plasma membrane. Extracellular proteins play roles in cell wall biogenesis and rearrangements of cell wall polymers including polysaccharides (glycoside hydrolases, esterases, lyases and expansins), lignins (peroxidases and laccases) and structural proteins (peroxidases).

##### Glycosyl transferases

Genes encoding enzymes belonging to several GT families such as GT2, GT8, GT31, GT34, GT47 and GT48, have detectable levels of transcripts in stems (see Additional file 1). Two genes of the GT2 family have high or moderate levels of transcripts (*CesA2*, *At4g39350*; *CesA5*, *At5g09870*). They were recently assumed to be associated with CesA1 (*At4g32410*) and CesA3 (*At5g05170*) in a tissue-specific way to form cellulose-synthase complexes [35]. *At3g07330 *and *At5g03760 *are cellulose synthase-like (CSL) proteins assumed to be involved in synthesis of non-cellulosic polysaccharides such as mannan, glucomannan and xylan [36,37].

Four out of 21 genes of the GT8 family have high or moderate levels of transcripts at intermediate stage of stem development (*At1g19300*, *GATL1*; *At5g15470*, *GAUT14*; *At2g20810*, *GAUT10*; *At3g18660*). *GATL1*, *GAUT14*, and *GAUT10 *have been predicted to encode proteins with putative galacturonosyltransferase activity, whereas *At3g18660 *is a glycogen starch initiation protein. Three genes of GT31 and GT34 families have high or moderate levels of transcripts. Enzymes in these families have not been experimentally characterized, but their putative functions are galactosyl transferases and xyloglucan 6-xylosyltransferases, and they could be involved in glycan processing [38]. In addition, the GT34 family was proposed to be involved in biosynthesis of galactomannan and xyloglucan [39,40].

Four genes of the GT47 family have high or moderate levels of transcripts at intermediate stage of stem development. *At2g28110 *(*FRA8*) encoding a putative glucuronyltransferase plays a role in secondary wall synthesis. *fra8 *mutants have short stems, thinner fiber walls with reduced amounts of glucuronoxylan in cell walls, and lack glucuronic acid residues in the xylans [31]. *At1g27440 *(AtGUT1) and *At5g61840 *(AtGUT2) were supposed to be involved in the synthesis of RG-II [41]. Recent reinvestigation of these transferases demonstrated that are rather related to the elongation of the backbone of xylan in stems (Alan Marchant, personnel communication). Finally, *At5g22940 *encodes a putative xyloglucan galactosyltransferase. Two highly expressed putative callose synthases (*At5g13000 *and *At1g05570*) from the GT48 family were identified.

Taken together, genes having high or moderate levels of transcripts identified in 5 GT families could be involved in the synthesis of cell wall polysaccharides, *i.e. *GT2, GT8, GT34, GT47 and GT48. Other functions include biosynthesis of glycan (GT31, GT34), and compounds involved in mobilisation of energy in the form of sucrose (GT8). Several genes were found to be homologous to poplar xylem-specific GT genes [42].

##### Extracellular proteins acting on carbohydrates

GHs belong to 12 different families whose functions were recently reviewed [4,26]. Fourty-five genes encoding proteins acting on carbohydrates were identified among which 42 are CAZys (Carbohydrate Active enZymes) and 6 are expansins. CAZys include glycoside hydrolases (GHs), carbohydrate esterases (CEs), and polysaccharide lyases (PLs) (see Additional file 3). About half of the identified GHs (24 genes) such as those belonging to GH1, GH3, GH9, GH16, GH27, GH28, GH31 and GH35 families could be involved in cell wall modification. Their substrates are assumed to be pectins (GH3, GH28, GH35), xyloglucans (GH1, GH16, GH31), or xylans, arabinoxylans and arabinans (GH3).

The most represented families are GH16, GH17, and GH1. Seven xyloglucan endotransglucosylase-hydrolases (XTHs) of the GH16 family were identified. Three of them (*At2g06850*, *At4g30290*, *At4g30270*) were expressed at high levels in young stems and at intermediate stage, but were down-regulated in mature stems. Some XTHs have been shown to function in cell elongation by loosening of cell walls [43]. It has also been shown that XTHs can be involved in formation of secondary cell walls of vascular tissues [44-46]. Seven genes of the GH17 family encoding putative β-1,3-glucanases were expressed at different stages of stem development. Only one was down-regulated in mature stems (*At3g07320*). Five of these enzymes are GPI-anchored. β-1,3-Glucanases catalyze the hydrolysis of β-1,3-glucan linkages of callose in plants, and play a role in various important physiological processes such as regulating pollen tube growth or defence against pathogen attack during fertilization [47]. Four genes of the GH1 family were identified by this transcriptomic analysis. *At1g61810 *and *At1g52400 *were up-regulated during stem growth whereas *At3g60130 *and *At3g18080 *were down-regulated. The enzymes of this family are known as β-glucosidases and are involved in diverse processes such as cell wall remodelling, formation of secondary walls, and activation of phytohormones [48].

The other GHs such as putative chitinases (GH19), β-D-galactosidases (GH35), and β-D-mannosidases (GH38) could be involved in post-translational modifications such as glycosylations of proteins [25]. It should also be noted that a mutation of *AtCTL1 *(GH19, *At1g05850*) causes ectopic deposition of lignin and aberrant shapes of cells with incomplete cell walls in the pith of inflorescence stems [49]. One enzyme of the GH31 family encodes an invertase (also called β-D-fructofuranoside-fructohydrolase) possibly involved in mobilisation of energy in form of sucrose [50].

**Figure 3:**
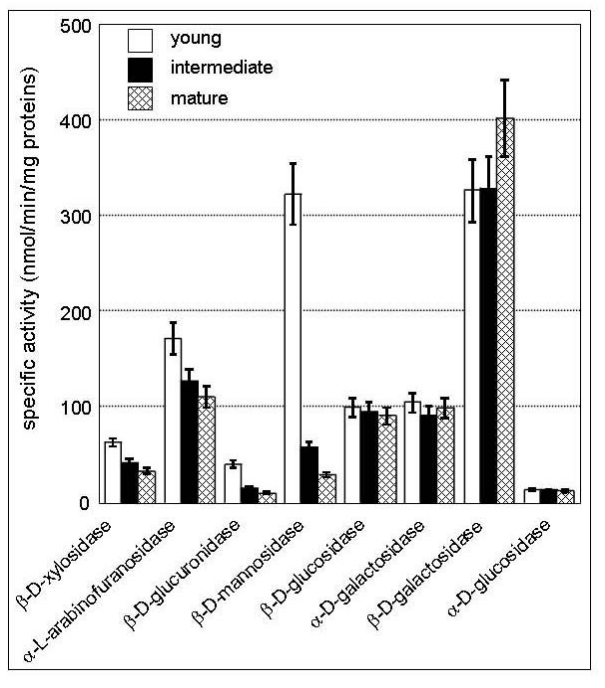
**Specific activities of several GHs**. All enzyme activities were measured *in vitro *at 37°C, using 50 μl of protein extracts and p-nitrophenyl-glycosides as substrates.

A previously published article demonstrated the presence of various exo-glycoside hydrolases in the mature stems [8]. Here, stems at three different stages of development were analyzed to determine the potential changes in the levels of activity of these enzymes. The results obtained (Figure 3) demonstrated the presence of all the GH activities tested at the three stages of stem development. Decreasing enzyme activities from young to mature protein extracts were observed for β-D-xylosidase, α-L-arabinofuranosidase, β-D-glucuronidase and β-D-manosidase. However, enzyme activities for β-D-glucosidase, α-D-galactosidase, β-D-galactosidase and α-D-glucosidase remained unchanged. This result as well as transcriptomic analyses (see Additional file 3) strongly suggested that the expression of GHs is regulated in relation to stem development.

Six carbohydrate esterases (CEs) were identified, these are the pectin methylesterases (PMEs) or pectin acetylesterases. Five of these genes were down-regulated in mature stems. Activities of these enzymes can cause major changes in the physical, chemical, and biological properties of pectins [51]. At acidic pH, demethylesterification by PMEs can promote the activity of polygalacturonases, thus contributing to cell wall loosening. On the contrary, PME activity was shown to be inversely correlated with the rate of growth of expanding tissues, suggesting possible involvement in wall rigidification. Two genes encoding polysaccharide lyases (PLs) were down-regulated either during the whole development of stems (*At3g07010*), or during the transition from young stems to intermediate stage (*At5g48900*). PLs catalyze the cleavage of pectate, the de-esterified product of pectins, which is the major component maintaining the structural integrity of cell walls in higher plants [2].

Five α-expansins and one β-expansin are expressed at high or moderate levels during stem development. Two α-expansin genes (*At2g28950*, *At2g40610*) were repressed during stem development whereas the gene encoding a β-expansin was up-regulated during the transition from intermediate stage to mature stems. Interaction of xyloglucans, xylans and other hemicelluloses with cellulose is modulated by expansins that presumably disrupt hydrogen bonds between these components [52].

##### Genes involved in the synthesis of monolignols

Monolignol biosynthetic pathway involves many different steps [53,54]. The first steps are part of the more general phenylpropanoid pathway including synthesis of soluble phenolics such as flavonoids and sinapic esters. Based on bioinformatics and functional studies, complete inventories of genes involved in this pathway have been published for *Arabidopsis *and a few genes of each multigene family were demonstrated to contribute to the phenylpropanoid pathway [54,55]. The level of transcripts of all these genes has been analyzed in the stem transcriptomes (see Additional file 2). Several genes were found to be expressed throughout stem development: hydroxycinnamoyl CoA:shikinate/quinase hydrocinnamoyl transferase (*HCT, At5g48930*), *p*-coumarate 3-hydroxylase 1 (*C3H, At2g40890*), ferulate 5-hydroxylase (*F5H, At4g36220*) and caffeic acid methyltransferase (*COMT, At5g54160*). Changes in the level of transcripts were observed for other genes. Level of transcripts of *PAL4 *was higher in young stems than in mature stems, whereas levels of transcripts of *PAL1 *and *PAL3 *increased at the mature stage. Among the four genes encoding the phenylalanine ammonia-lyase (PAL), only *PAL1*, (*At2g37040*), *PAL2 *(*At3g53260*) and *PAL4 *(*At3g10340*) have been shown to be important in the phenylpropanoid pathway [56]. Concerning the next step of the pathway, level of transcripts of the trans-cinnamate 4-hydroxylase gene (*C4H, At2g30490*) was found to be higher in mature stems. Several 4-coumarate CoA ligase genes (*4CL1*, *At1g51680*; *4CL2*, *At3g21240*; *4CL3*, *At1g65060*; *4CL 4, At3g21230*) were shown to be involved to lignification [57]. The increase of the level of transcripts of *4CL2 *during stem development could be related to this process. In contrast, nothing is known about the function of *4CL-like1 *(*At1g20510*), whose level of transcripts was found to be modulated during stem development. The levels of transcripts of caffeoyl-CoA reductase genes (*CCoAOMT-3*, *At3g61990*; *CCoAOMT-7*, *At4g26220*) strongly increased during stem development. However, no hypothesis on their role in lignification could be proposed since only *CCoAOMT1 *(*At4g34050*) has been shown to be related to lignification [58]. The two last steps of the pathway are specific to lignification [53]. They involve cinnamoyl CoA reductase (*CCR*) and cinnamyl alcohol dehydrogenase (*CAD*) genes. Level of transcript of *CCR1 *(*At1g15950*) was higher at the young elongating stems. Studies using knockout mutants showed that *CCR1 *is the major expressed gene in the biosynthetic pathway of lignin [59,60]. Nine genes belong to the *CAD *multigene family [54,55]. The levels of transcripts of three *CAD *genes (*CAD1*, *At4g39330*; *CAD6*, *At4g34230*; *CAD7*, *At2g21730*) decreased during the transition from young elongating stems to mature stems. Only *CAD2* (*At3g19450*) and *CAD6 *have been shown to play major role in lignification [61]. A minor role of *CAD1 *in lignification of elongating stems has been shown [62]. The level of transcripts of all the other *CAD *genes which have not been demonstrated to be linked to lignification [62] remained the same throughout stem development. In conclusion, most genes already shown to contribute to monolignol biosynthesis were found to be expressed in stems. A few of them like *PAL4 *and *4-CL2 *showed increase in their level of transcripts during stem development as expected from their demonstrated roles in monolignol biosynthesis. However, the function of genes such as *PAL3*, *4CL-like1*, *CCoAOMT-3*, *CCoAOMT-7 *and *CAD7 *should be investigated since their level of transcripts is modulated during stem development. The genes identified as highly expressed in stems are the same as those reported in previous studies [19,28] although the sampling and the growth conditions of the plants were different. This demonstrates the importance of these genes in the monolignol metabolic pathway. In addition, expression data bases such as GeneCAT  using Affimetrix information and RT-PCR [54-62] indicate the same trend, that is high expression in the first and the second internodes.

##### Extracellular oxido-reductases

Four genes encoding peroxidases are expressed at moderate or high levels in developing stems (*At2g37130*, *At4g21960*, *At4g33870*, *At5g64120*). One of them is up-regulated in mature stems (*At4g33870*) whereas one is down-regulated (*At5g64120*). In addition, 14 peroxidase genes have low levels of transcripts at the three stages of stem development analyzed. This study identified peroxidase genes that were not previously shown to be differentially expressed in developing stems [28]. Peroxidases belong to a large multigene family of 73 members in *Arabidopsis *and play many roles during development and in response to environmental changes [63]. Two peroxidases of *Arabidopsis *were shown to play a role in promoting root elongation [64]. Conversely, peroxidases can cause reduction in cell wall extensibility by formation of diferuloyl bridges between pectin residues, isodityrosine bridges between hydroxyproline-rich proteins like extensins, and covalent links between lignin precursors [65,66].

Eight genes encoding proteins with predicted multicopper oxidase domains have been identified. Such enzymes catalyse full four-electron reduction of dioxygen (O_2_) to water (H_2_O) using a variety of substrates [67]. Four genes encoding putative laccases have moderate or high level of expression in developing stems (*At2g38080*, *At5g03260*, *At5g05390*, *At5g60020*). Laccase activity in plants has been assumed to participate in cell wall lignification [68]. All four genes are up-regulated during the transition from young stems to intermediate stage as previously described [28]. The expression of these genes was also reported to cluster with most monolignol biosynthetic genes in stems [28]. Three genes encoding multicopper oxidases of the SKS family were identified (*At4g12420*, *At1g76160*, *At1g41830*). Two of them were down-regulated during stem development (*At4g12420*, *At1g76160*). It was shown that SKU5 (*At4g12420*) is involved in the control of root growth [69], and that SKS6 (*At1g41830*) contributes to cotyledon vascular patterning during development [70].

Three genes encoding germin-like proteins (GLPs) were expressed at high levels in young stems and at intermediate stage of development. Two of them were strongly down-regulated in mature stems (*At1g72610*, *At5g20630*). Although the functions of GLPs are not clearly understood, it was shown that a GLP gene is highly expressed during cotton fiber elongation, but is repressed once the rate of growth slows down [71].

Considering the variety of gene families involved in modification of carbohydrates and in oxido-reduction reactions, these results show that all cell wall components probably undergo modifications during stem growth. Even if many of these gene families were known to be involved in cell wall assembly or remodelling, this transcriptomic analysis allows the identification of genes involved in stem development processes.

#### 2.3. Genes encoding secreted proteins involved in other cell wall functions

##### Proteins with interaction domains

Several families of genes encoding proteins with putative domains of interaction with polysaccharides and/or proteins were identified. These include proteins with leucine-rich repeat (LRR) domains (6 genes), lectins (7 genes) and enzyme inhibitors (8 genes). Due to their high specificity towards carbohydrates, lectins may be involved in various physiological functions [72]. They could be involved in the recognition between cells or between cells and various carbohydrate-containing molecules or in assembly of the polysaccharide matrix. Other possible functions of lectins in plants include: transport and packaging of carbohydrates, and mobilization of storage materials. Inhibitors of PMEs regulate plant PME activity. Five genes encoding PME inhibitors were identified among which 3 are down-regulated in mature stems (*At5g64620*, *At5g62350*, *At4g25260*). This down-regulation seems to be correlated with that of 3 genes encoding PMEs mentioned above.

##### Proteins involved in signaling

Nine genes encoding arabinogalactan proteins (AGPs) and 7 genes encoding fasciclin-like AGPs (FLAs) had high or moderate levels of transcripts in developing stems. In addition, 6 and 4 genes encoding AGPs and FLAs, respectivement, had low levels of transcripts. Five FLA genes are down-regulated in mature stems. AGPs belong to a class of Hyp-rich glycoproteins that are highly glycosylated and abundant in plant cell walls [27]. AGPs have been involved in plant development such as cell fate determination, somatic embryogenesis, and cell proliferation [73]. AGPs are assumed to be signal molecules [74]. The association of AGPs with pectic polysaccharides has also been suggested [75]. FLAs constitute a distinct class of proteins. Proteins containing fasciclin domains have been shown to function as adhesion molecules in a broad spectrum of organisms [76]. More recently, AGPs and FLAs were found to be associated with wood formation in poplar [77].

Twenty-two genes encoding putative receptor kinases are expressed at high or moderate levels in developing stems. Only a few of them are up- or down-regulated. Protein kinases play a very significant role in signal transduction. Our screening revealed that 14 genes are LRR-RLKs [78]. Their extracellular domain is composed of tandem repeats of a well-conserved leucine-rich motif and their intracellular domain consists in a protein kinase with Ser/Thr specificity. LRR-RLKs are involved in development, hormone perception, and pathogen response [79].

##### Proteases

Twenty-four genes encoding putative proteinases were identified. These enzymes belong to Asp-, Ser- and Cys-protease families. Proteinases may be involved in processing and/or turnover of CWPs, as well as in peptide signal transduction [80,81]. Several proteomic analyses of plant cell walls show a large discrepancy between the observed and the expected molecular weights of proteins, suggesting the existence of a turnover of CWPs [8,12,82]. Expression of a Cys protease was shown to be associated with programmed cell death [83]. The occurrence of many proteases and their high expression levels during stem development suggests that these enzymes are actively involved in cell expansion and/or in secondary wall formation. However, their activity could be modulated by the presence of many protease inhibitors since 14 genes encoding such inhibitors were found to have moderate or high levels of transcripts.

##### Structural proteins

Only a few genes encoding structural CWPs were found to have moderate or high levels of transcripts in developing stems. This low number might be the consequence of the difficulty to design specific probes for genes encoding repetitive sequences of amino acids such as structural CWPs. Indeed, CATMA does not offer a good coverage of such gene families, *e.g. *only 5 extensin genes out of 19 were analyzed. Identified genes belong to the major classes of cell wall structural proteins, *e.g. *extensins, Proline Rich-Proteins (PRPs), Glycine Rich-Proteins (GRPs) and LRR-extensins (LRXs). Five out of thirteen genes are down-regulated in mature stems whereas two genes encoding GRPs are up-regulated (*At2g05380*, *At2g05520*). Structural CWPs such as extensins and PRPs are assumed to be insolubilized when cell elongation ceases [63,84]. Extensins, GRPs, and PRPs were shown to be expressed in specific cell types including xylem and phloem tissues by tissue-printing of stems [85]. The role of GRPs in the building of the cell walls of protoxylem elements has been shown by immunological studies [86].

##### Miscellaneous proteins

A number of genes (59 in total) encoding miscellaneous proteins were identified by transcriptomic analysis. A large proportion of these genes encode proteins predicted to be protease inhibitors (14 genes) or are involved in lipid metabolism (12 genes). Lipids are essential for cell growth [87] and results of these analyses suggest that lipid metabolism is important for stem development. Five genes encoding thaumatin-like proteins among which 3 are repressed during stem development were identified. They encode pathogenesis-related proteins that are expressed constitutively at certain stages of plant development [88]. Identification of several Zn transporters suggests that Zn is an essential micronutrient for stem development. Four genes encode phytocyanins (*At1g64640*, *At4g12880*, *At5g15350*, *At1g72230*). Three of these genes are down-regulated in mature stems. It should be noted that 14 genes of the same family are expressed at low levels in developing stems. Phytocyanins are assumed to play a role in oxido-reduction processes in cell walls as electron transfer proteins [89]. In addition, stellacyanins and uclacyanins have structural domain possibly interacting with structural CWPs.

##### Proteins of yet unknown function

Numerous genes (119) encoding proteins with unknown function were identified in this transcriptomic analysis. Some of which share structural domains or the so-called domains of unknown function (DUF). About half of them encode proteins predicted to have trans-membrane domains or GPI anchors. The encoded proteins may play house-keeping functions or be specifically involved in stem development.

Altogether, many gene families encoding secreted cell wall proteins were found to have members expressed at high or moderate levels during stem development. Proteinase genes are actively transcribed, suggesting essential roles in protein processing, turnover, or signalling. Finally, many genes have yet unknown function during stem development.

##### Validation of expression microarray results by Real-Time-qPCR

In order to validate the results from microarray analysis, transcripts of 13 selected genes, which were significantly expressed in the microarray studies, were analyzed by Real-Time-qPCR (see Additional file 4). The majority of these selected genes were related to cell wall biogenesis. These included 4-coumarate-CoA ligase (*4CL 2*), caffeoyl-CoA 3-O-methyltransferase (*At4g26220*), cellulose synthase subunit (*IRX 5*), cellulose synthase, catalytic subunit (*At5g44030, IRX5*), cellulose synthase, catalytic subunit (*At5g09870*), putative laccase (*At5g60020*), GH of family 19 (*At3g16920*), pectate lyase (*At4g24780*), phosphate responsive protein (*At5g51550*), plasma membrane intrinsic protein 2C (*At2g37180*), putative expansin (*At2g40610*), chlorophyll A-B binding protein (*At2g34430*), catalase 2 (*At4g35090*) and putative raffinose synthase (*At5g20250*). Two genes, actin 2 (*At3g18780*) and 26S proteasome regulatory subunit (*At4g24820*), were selected as endogenous control. Compared with transcriptomic analyses, significant correlation was confirmed by RT-qPCR according to the calculated average in transcript levels of all the tested genes.

### 3. Transcriptomics vs proteomics

In order to better understand gene regulation in stems, data from transcriptomics were compared to those of a previous proteomic study performed using the same biological material, *i.e. *mature *Arabidopsis *stems [8]. This proteomic study was designed to identify *N*-glycoproteins trapped on ConA sepharose and allowed the identification of 90 genes encoding secreted proteins. Their levels of transcripts were searched in the stem transcriptome. Results were obtained for 72 genes on the CATMA hybridized with total RNAs from mature stems (see Additional file 5). Eighteen genes were found to have levels of transcripts lower than background, 42 had weak levels of transcripts, 5 moderate levels of transcripts and 7 high levels of transcripts. Altogether, 25% of the genes identified through proteomics have levels of expression below background and 58% have low level of transcripts. The percentage of genes having detectable levels of expression decreases from about 82% to 75% during stem growth. The encoded proteins might have long half-lifes and remain in cell walls after the disappearance of the mRNAs. It means that transcriptomics fails to give an overall picture of proteins present in cell walls at a given time.

Conversely, only 25 genes identified through proteomics are found among the 345 genes encoding secreted proteins expressed at high or moderate level. This means that many proteins escaped this proteomic analyses. One explanation might be the partial analysis of proteins separated by 2-D electrophoresis [8]. In addition, low abundant proteins are not usually identified in proteomic studies, and structural proteins are difficult to extract from cell walls since they form insoluble networks [84]. Proteins with transmembrane domains are also not easily extracted by salts. AGPs and FLAs are difficult to identify because of their high level of glycosylation. However, these explanations are not valid for proteins like GHs that are soluble. Alternatively, the numerous proteases present in cell walls probably play a great role in protein turnover, although it was shown that glycoproteins are more resistant against digestion by proteases [90]. In addition, some regulatory mechanisms preventing the translation of certain mRNAs may also exist. Such discrepancies between proteomics and transcriptomics have been described in yeast [91]. Finally, proteome and transcriptome approaches appear to be complementary to get an overview of genes expressed in developing stems. The observed discrepancies show the importance of post-transcriptional regulations.

## Conclusion

Here we have examined the expression patterns of the genes encoding cell wall proteins. Some of the genes identified in this work were previously identified by various microarray analyses of *Arabidopsis *stems. These include XTH [19,92], 1,3-β-glucanase and pectin esterase [18], pectate lyase, AGP, glycine rich and proline rich proteins [19]. However, we identified and established expression patterns of many new genes possibly involved in primary and secondary cell wall biogenesis. Many genes involved in the synthesis of cell wall components were found to be transcribed as well as many genes encoding secreted proteins. The latter genes encode proteins acting on carbohydrates, oxido-reductases, proteins with interaction domains and structural proteins. Among secreted proteins, proteins acting on carbohydrates represent the most abundant class after proteins of unknown function. In addition, genes encoding proteins such as AGP, FLAs, GRPs and PRPs not reported by proteomic analyses, had high levels of transcripts. This transcriptomic analysis allowed identification of many gene candidates that play a role in cell wall construction during stem development. Both primary and secondary walls are drastically modified thus allowing cells to undergo elongation during early steps of development. These modifications are part of the differentiation processes during late stages of development. The enzymes which catalyze glucan-chain elongation in cellulose, belonging to the family 2 of glycoside transferase (GT2), are crucial for primary and secondary wall formations [35,93-95]. In plants this family of GTs, designated as the cellulose synthase catalytic subunits (CESA), has multiple members. In the present work several highly expressed genes of this family of enzymes have been identified (see Additional file 1). Comparison of the gene expression profile of CESA genes obtained in the present study with those of GeneCAT database () showed similar high expression profile of 7 genes with exception of *At2g25540 *which is not expressed according to GeneCAT database. Though good correlation in expression scores of these genes were obtained, some differences were observed concerning expression level or difference in expression. These differences could be consequences of developmental stage of plant, condition of growth, environment and from minor unavoidable experimental errors during extraction of RNAs and measurement. Finally, comparison of transcriptomic and proteomic analyses showed that about 25% of the genes encoding proteins identified by proteomics had level of transcripts above background in the CATMA analysis. Conversely, only a small proportion of genes identified by proteomics were identified by transcriptomics, *i.e. *only 25 out of the 339 genes encoding secreted proteins were expressed at high or moderate levels. Such discrepancies between proteomics and transcriptomics suggest complex regulatory mechanisms which occur at post-transcriptional levels.

## Methods

### Plant material

Wild-type *Arabidopsis thaliana*, Wassilewskija ecotype, was grown in the greenhouse at 20°C to 22°C with a 16 h-photoperiod at 150 *μ*E.m^-2^.s^-1^. Three different stages of plant development were used: young elongating stems at stage 5.10 (3–5 cm), intermediate stage 6.10 (8–12 cm), and mature stems at stage 6.90 (20–24 cm) according to Boyes et al. [96]. Three times 30 plantlets were collected for each experiment.

### Isolation of xylans

290 mg of *Arabidopsis thaliana *stems were ground and then extracted sequentially with 80% ethanol and boiling ammonium oxalate (twice) for 2 hours, and then with 1 M and 4 M KOH overnight at 4°C. Extracts were dialysed extensively against water and then lyophilised.

### Sugar composition in various stages of stem development

Ethanol-insoluble stem residues were hydrolyzed using trifluoroacetic acid (2 M, 2 h at 110°C), followed by an 18 h methanolysis at 80°C with dry 2 M methanolic-HCl. The resulting methyl glycosides were then converted into their TMS-derivatives and separated by gas chromatography (GC) with helium as carrier gas, equipped with a flame ionization detector and a WCOT fused silica capillary column (length 25 m, i.d. 0.25 mm and film thickness 0.4 μm) with CP-Sil 5 CP as stationary phase. The standard deviation values were determined from three replicate assays.

### High performance anion exchange chromatography of xylan oligosaccharides

Hydrolysis of 1 mg of the xylan fractions in 0.5 mL of 10 mM NaOAc pH 5.5 was performed using 4.5 units of endo-(1 → 4)-β-D-xylanase (Megazyme International Ireland, β-xylanase M6) at 28°C overnight. Enzymatic hydrolysis was stopped through ethanolic (95%) precipitation (final volume 2.5 mL). Solutions were centrifuged for 10 min at 5500 rpm (Bioblock Scientific, Sigma 3K12). Supernatants were freeze-dried then suspended in water (1 mL). Xylanase-generated fragments were analysed by a HPAEC system (Dionex X500) equipped with a CarboPac PA-1 column combined with pulse amperometric detection (PAD). Samples (20 μL) were eluted at 1 mL min^-1 ^with the following; NaOAc gradient in 100 mM NaOH: 0 → 5 min, linear gradient of 0 → 5 mM NaOAc; 5 → 20 min, linear gradient of 5 → 30 mM NaOAc; 20 → 42 min, 30 mM NaOAc isocratic step until 45 min. Each elution was followed by a wash with 1 M NaOAc in 100 mM NaOH, and subsequent equilibration for 5 min with 100 mM NaOH.

### Glycoside hydrolase activities

Preparation of protein extracts from stems of *Arabidopsis *and the activity of various GHs were determined under the standard conditions previously described [8]. The standard conditions were 2 mM *p*NP-glycosides (Sigma Chemical Co., St Louis, MO, USA), 0.1 M acetate buffer (pH 5.0), 2 mM sodium azide, and 50 μL of protein extract in a total volume of 0.5 mL. The reaction was carried out at 37°C for 5–60 min (depending on activity) and stopped by the addition of 0.5 mL 0.4 M sodium chloride. Controls were stopped at time 0. Concentration of the resulting *p*NP was determined spectrophotometrically at 405 nm by comparison with a calibration curve. The standard deviation values were determined from three replicate assays.

### RNA extraction

For each samples, 2 g of material was frozen in liquid nitrogen and ground to powder. One hundred mg of this powder was transferred in an Eppendorf tube and 1 mL of TRIZOL^® ^reagent (Invitrogen, Carlsbad, CA) was added. The homogenate was vortexed for15 s, kept at room temperature for 5 min, then centrifuged for 15 min at 12000 *g*. The supernatant was transferred into a new tube and 200 μL of chloroform was added. After 15 s of shaking, the tube was centrifuged for 15 min at 12000 *g*. Total RNAs from the resulting upper aqueous phase was then precipitated with 500 μL of isopropanol and centrifuged at 12000 *g *for 20 min. The pellet obtained was washed with ethanol (70%) and resuspended in 20 μL of DEPC-treated water. The average concentration of RNAs was about 500 ng/μL. RNA integrity was checked with the Agilent Bioanalyzer (Waldbroon, Germany).

### Transcriptome studies

Microarray analysis was carried out at the Unité de Recherche en Génomique Végétale (URGV, Evry, France), using the CATMA array [33,34], containing 24,576 gene-specific tags from *Arabidopsis*. The GST amplicons were purified on Multiscreen plates (Millipore, Bedford, USA) and resuspended in TE-DMSO at 100 ng/μL. The purified probes were transferred to 1536-well plates with a Genesis workstation (TECAN, Männedorf, SW) and spotted on UltraGAPS slides (Corning, New York, USA) using a Microgrid II (Genomic Solution, Huntingdon, UK). The current CATMA version printed at the URGV consists of three metablocks, each composed of 64 blocks of 144 spots. A block is a set of spots printed with the same print-tip. In these arrays, a print-tip is used three times to print a block in each metablock. RNA samples for a condition were prepared by pooling RNAs from 30 plants. For each comparison, one dye-swap was performed as a technical replication with fluorochrome reversal (i.e. two hybridizations per comparison). cRNAs were produced from 2 μg of total RNA from each pool with the "Message Amp aRNA" kit (Ambion, Austin, TX). Then 5 μg of cRNAs were reverse transcribed in the presence of 200 u of SuperScript II (Invitrogen, Carlsbad, CA), cy3-dUTP and cy5-dUTP (NEN, Boston, MA) for each slide as described in Lurin et al. [97]. 30 pmol. of each labelled target sample per slide were combined, purified and concentrated with YM30 Microcon columns (Millipore, Bedford, MA) prior to hybridization. Slides were pre-hybridized for 1 h and hybridized overnight at 42°C in 25% formamide. Slides were prehybridized for 1 h and hybridized overnight at 42°C in 25% formamide. Slides were washed with 2× SSC+ 0.1% SDS 4 min, 1× SSC 4 min, 0.2× SSC 4 min, 0.05× SSC 1 min and dried by centrifugation. The arrays were scanned on a GenePix 4000A scanner (Axon Instruments, Foster City, USA) and images were analysed by GenePix Pro 3.0 (Axon Instruments, Foster City, USA).

### Statistical analysis of microarray data

Experiments were designed with the statistics group of the URGV. Statistical analysis was based on one dye swap (*i.e. *two arrays, each containing 24,576 GSTs and 384 controls) as described in Lurin et al. [96]. Controls were used for assessing the quality of the hybridizations, but were not included in the statistical tests or the graphic representation of the results. For each array, the raw data comprised the logarithm of median feature pixel intensity at wavelengths 635 (red) and 532 nm (green). No background was subtracted. In the following description, log ratio refers to the differential expression between two conditions. It is either log_2 _(red/green) or log_2 _(green/red) according to the experimental design. Array-by-array normalization was performed to remove systematic biases. First, we excluded spots that were considered to have badly formed features. Then, we performed global intensity-dependent normalization using the LOESS procedure to correct the dye bias. Finally, for each block, the log_2 _ratio median calculated over the values for the entire block was subtracted from each individual log ratio value to correct print tip effects on each metablock. To determine differentially expressed genes, we performed a paired t test on the log ratios, assuming that the variance of the log ratios were the same for all genes. Spots displaying extreme variance (too small or too large) were excluded. The raw P values were adjusted by the Bonferroni method, which controls the Family Wise Error Rate (FWER). The genes with an FWER lower than 5% were considered to be differentially expressed. We used the Bonferroni method (with a type I error equal to 5%) in order to keep a strong control of the false positives in a multiple-comparison context [98].

### Background estimation

After the normalization procedure described above, we obtained normalized ratios and intensities per sample for each dye swap. The range of the normalized log_2 _intensities per sample was from 0 to 16. In these data, background was not subtracted, thus we had to estimate a background level for each dye swap to get information about the hybridization level. In most transcriptomic analysis, the background is calculated by the scanner software from the pixels intensities in the spots vicinity, which underestimates dramatically the real background on the spots. For example it does not take into account the autofluorescence of the spotted DNA. Here we decided to calculate a more accurate background based on the intensity value of 1220 negative spots. These negative spots corresponded either to negative human probes or *Arabidopsis *probes for which the hybridization signal remained at the lowest level in more than 3000 CATMA results obtained on the URGV platform. These genes could be either very poorly expressed under the sensitivity of the array, or expressed in very specific tissues or environmental conditions. The estimated background is the sum of the average log_2 _intensity value of the "negative spots" plus twice their standard deviation. This value was compared to the normalized log_2 _intensity to get an estimation of the hybridization signal above background.

### Data deposition

Microarray data from this article was deposited at Array-Express (; accession E-MEXP-802) and CATdb (; Project AF09-Lignin) according to the "Minimum Information About a Microarray Experiment" standards.

### Quantitative real-time RT-PCR validation

Quantitative real-time RT-PCR validation was performed for 13 genes on the samples described in the microarray section. The primers for RT-PCR were selected with Primer3 (, optimal length 21 nt, optimal temperature 60°C) (see Additional file 6). The primer pairs were first tested on a dilution series of genomic DNA (5 ng, 0.5 ng, 0.05 ng, 0.005 ng) to generate a standard curve and assess their PCR efficiency, which ranged between 90% and 99%. Reverse transcription was performed on 1 μg of total RNA with an oligodT primer (18 mer) and the Superscript II reverse transcriptase (Invitrogen, Carlsbad, CA), for 1 hr at 42°C in 40 μL. The enzyme was then heat inactivated at 65°C and the samples were treated with RNase H. Quantitative PCR reactions were performed in 15 μl, with 0.1 μl ng of RT reaction, 900 nM final concentration of each primer pair, and SYBRGreen PCR master mix 2X (Eurogentec, Seraing, Belgium). Corresponding minus RT controls were performed with each primer pair. Conditions were as follows: 95°C, 10 min; 403 (95°C, 15 s; 60°C, 1 min) and a dissociation step, to discriminate primer dimers from the PCR product. All reactions were performed in RT duplicate with the ABI PRISM 7900 HT Sequence Detection System (Applied Biosystem, Pleasanton, CA) and data were analyzed with the SDS software provided by the manufacturer. Two housekeeping genes were used to calculate the average normalisation factor: *At3g18780* and *At4g24820* for each sample pairs. Then normalized ΔCT for each differentially expressed gene were calculated as following: Norm ΔCT = raw ΔCT – Norm. Factor.

### Bioinformatics analyses

The presence of signal peptides, GPI-anchors and transmembrane domains was predicted using PSORT , TargetP  and Aramemnon . Annotation of transcription factors was done according to NCBI . Functional annotation of secreted proteins was done using InterProScan . Annotation of GTs was done according to the Cell Wall Genomics site of the Purdue University  and to the CAZy nomenclature . Annotation of proteins involved in the synthesis of monolignols (lignin toolbox) was done according to Raes et al. [54].

## Abbreviations

AGP: arabinogalactan protein; CAZy: Carbohydrate Active Enzyme; CE: carbohydrate esterase; CWP: cell wall protein; GH: glycoside hydrolase; GLP: germin-like protein; GPI: glycosylphosphatidylinositol; GRP: glycine-rich protein; PTM: post-translational modification; GT: glycosyltransferase; LRR: leucine-rich repeat; PL: polysaccharide lyase; PME: pectin methylesterase; PMEI: pectin methylesterase inhibitor; PRP: proline-rich protein; RLK: receptor-like kinase; SAM: shoot apical meristem; XTH: xyloglucan endotransglucosylase/hydrolase.

## Authors' contributions

ZM performed preparation of samples for analyses as well as designed the experiments, interpreted the results, wrote the manuscript, and made final revision. EJ conducted the classification of genes, provided intellectual input on interpretation of the data and editing and revising the manuscript. HSC performed bioinformatic analysis of the microarray data. DO helped in analyses of some results. JPR and CP participated in the microarray experiments and provided the statistical analyses of the microarray data. SP provided results of quantitative real-time RT-PCR. CR and PL provided results of sugar composition in various stages of stem development and determined the xylan structure. LJ supervised organization of the manuscript, provided critical analyses of the data, and gave final approval of its readiness for submission. All authors read and approved the final manuscript.

## Supplementary Material

Additional file 1 **Level of transcription of genes encoding glycosyl transferases in stems**. The data show expression levels of the genes encoding predicted glycosyl transferases at three different developmental stages: (d2) young elongating stems at stage 5.10 (3–5 cm), (d10) intermediate stage 6.10 (8–12 cm), and (mature) mature stems at stage 6.9 (20–24 cm) according to Boyes *et al. *[96]. Only genes having level of expression higher than background are listed. Glycosyltransferases have been classified into families, according to the CAZy database . Levels of expression are expressed as log2 of signal mean intensity. Click here for file

Additional file 2 **Level of transcription of genes encoding proteins putatively involved in synthesis of lignin monomers in stems**. This table shows CATMA microarray analysis of genes involved in the lignin pathway. All the genes belong to the so-called "lignin toolbox" as defined by Raes *et al. *[54]. Stems were analyzed at three different stages of development: (d2) young elongating stems at stage 5.10 (3–5 cm), (d10) intermediate stage 6.10 (8–12 cm), and (mature) mature stems at stage 6.9 (20–24 cm) according to Boyes *et al. *[96]. Levels of expression are expressed as log2 of signal mean intensity. Click here for file

Additional file 3 **Genes encoding secreted proteins expressed at a moderate or high level in stems at three different stages of development**. The data provide level of transcripts of genes encoding secreted proteins at a moderate or high level in stems. Stems were analyzed at three different stages of development: (d2) young elongating stems at stage 5.10 (3–5 cm), (d10) intermediate stage 6.10 (8–12 cm), and (mature) mature stems at stage 6.9 (20–24 cm) according to Boyes et al. [96]. Levels of expression are expressed as log2 of signal mean intensity. Subcellular localization was predicted using PSORT , TargetP  and Aramemnon . Functional domains were predicted using InterProScan . Only PFAM(PF) and Prosite (PS) domains are mentioned. Click here for file

Additional file 4 **Validation of microarray data using RT-qPCR analysis of some selected genes**. Thirteen selected genes, which were significantly expressed in the microarray studies, were analyzed by Real-Time-qPCR. Analyses were performed using stems at three different stages of development: (d2) young elongating stems at stage 5.10 (3–5 cm), (d10) intermediate stage 6.10 (8–12 cm), and (mature) mature stems at stage 6.9 (20–24 cm) according to Boyes et al. [96]. Average in transcript levels were calculated and were compared for all the tested genes obtained form Real-Time-qPCR and CATMA microarray analysis. Click here for file

Additional file 5 **Levels of expression of genes encoding cell wall proteins identified through proteomics of stems**. The data from transcriptomics are compared with those of a previous proteomic study performed using the mature *Arabidopsis *stems [8] CATMA microarray analysis of stems were performed using three different stages of development: (d2) young elongating stems at stage 5.10 (3–5 cm), (d10) intermediate stage 6.10 (8–12 cm), and (mature) mature stems at stage 6.9 (20–24 cm) according to Boyes *et al. *(2001). Proteomic data are from Minic *et al. *[8]. Levels of expression are expressed as log2 of signal mean intensity. Only statistically significant differences are indicated (p-value < 0.05). Functional domains were predicted using InterProScan . Only PFAM (PF), Prosite (PS) and some IPR (Interpro) domains are mentioned. Click here for file

Additional file 6 **Oligonucleotide primers used for real-time quantitative PCR**. This table represents the sequences of gene-specific primers derived from nucleotide sequences of selected genes. Click here for file
